# The use of technology-assisted intervention in vocabulary learning for children with autism spectrum disorder: a systematic review

**DOI:** 10.3389/fpsyg.2024.1370965

**Published:** 2024-05-15

**Authors:** Ana Lucia Urrea, Vanessa Fernández-Torres, Isabel R. Rodriguez-Ortiz, David Saldaña

**Affiliations:** Laboratorio de Diversidad Cognición y Lenguaje, Departamento de Psicología Evolutiva y de la Educación, Universidad de Sevilla, Seville, Spain

**Keywords:** autism spectrum disorder, technology, intervention, vocabulary, systematic review

## Abstract

**Introduction:**

Children with autism spectrum disorder may show delays in vocabulary development. Technology-based interventions could facilitate the teaching of different vocabulary skills; however, it is still not clear whether technology represents an added value.

**Methods:**

The current review preregistered in PROSPERO evaluates the efficacy of technology-based interventions in vocabulary learning for children with autism spectrum disorder. We selected articles published in the period 2006–2022 from five databases.

**Results:**

The results identified two group studies, one within subject design, nine single-case studies and one randomized controlled design in participants aged 0–16 years who had used technological devices to learn vocabulary. Overall, five of the 13 studies showed positive results of using technology-assisted intervention, six described mixed results, one described negative result, and one described no differences in technology-assisted intervention. The studies are divided into the categories of efficacy of technology and comparison between technology and non-technology.

**Discussion:**

In summary, technology, such as tablets and computers, might be useful tools to improve vocabulary skills in certain children with ASD. However, the various degrees of impact found in the studies we reviewed indicate that personalized assessments, acknowledgment of previous experiences, and awareness of the context of usage are essential. The contrast with nontechnological approaches highlights the necessity for more detailed studies to pinpoint the precise conditions under which technology-based interventions can offer the most advantages.

**Systematic review registration:**

[https://clinicaltrials.gov/], identifier [CRD42021238758].

## Introduction

Children with autism spectrum disorder (ASD) typically have impaired verbal and nonverbal communication (American Psychiatric Association, 2013). Some children with ASD present a delay in language acquisition compared to typically developing children (Landa, 2007; Lauritsen, 2013). According to Tager-Flusberg and Kasari (2013), half of the population with autism do not develop useful speech by the age of three and they may develop fluent speech after the age of 4 years. Ellis Weismer et al. (2010) and Wodka et al. (2013) evaluated children with ASD and found that only 3% of the population had normal levels of language compared to typically developing children; the rest of the sample with ASD presented delays in language acquisition. Furthermore, specifically for vocabulary development, studies have shown that children with ASD have lower levels of expressive and/or receptive vocabulary compared to the typical population (Kwok et al., 2015; Belteki et al., 2022). A weakness in receptive language has been found very early in the development of children with ASD (Ellis Weismer et al., 2010). For receptive vocabulary, some children with ASD may have relatively significant deficits, even when expressive language appears to be moderately intact (Davis et al., 2016). Furthermore, Ellis Weismer et al. (2021) found that toddlers with ASD aged 24–36 months had significantly higher expressive language age-equivalent scores than receptive language age-equivalent scores on two different assessment measures. Regarding expressive language, in a study by Smith et al. (2007) including children with ASD and children without ASD, expressive vocabulary was evaluated at specific points in life, reporting that children with ASD showed lower expressive vocabulary and delays in first utterances compared to those with typical language development.

### Methods for vocabulary learning in children with ASD

Many different types of intervention methods have been developed to optimize language development, including vocabulary, in children with ASD, although the evidence base for their impact on vocabulary acquisition is varied and still in need of further research (Donolato et al., 2023). Methods vary in how explicit the goals of the intervention are for the participants. They range from incidental and implicit methods grounded in more developmental constructive theories to explicit instruction, in which words are modeled and their learning specifically reinforced. In between, hybrid approaches attempt to provide this explicit structure in naturalistic or quasi-naturalistic communicative situations.

Explicit methods used in autism include, among others, the picture exchange communication system (PECS) and many applied behavior analysis (ABA) approaches (Rhea, 2008; Will et al., 2018). PECS uses pictures or symbols combined with behavioral strategies to teach the child how to use the images in a functional way to request what he or she desires. This method has shown promising results in facilitating communication in children with ASD (Bottema-Beutel et al., 2019). ABA (Fisher et al., 2013) has been extensively applied to vocabulary intervention. In ABA, each step is taught one by one, presenting specific models, and using prompts followed by reinforcement of appropriate responses. Discrete trial intervention (DTI), based on ABA, uses strategies such as shaping, prompting, prompt fading, and reinforcement.

Contemporary ABA approaches attempt to include these strategies in naturalistic situations, this improving generalization. They include what are known as milieu teaching methods, such as prompt-free training, incidental teaching, and mand-modeling (Rhea, 2008).

Social-pragmatic strategies focus on facilitating communication and language learning within meaningful communicative interactions led by the child. One of these methods and programs is the floor time or the developmental, individual-difference, relationship-based (DIR) model. The Hanen method or Pediatric Autism Communication Therapy (PACT) aims to train parents to optimize their communication strategies in these contexts.

Other language interventions focus on language precursors, such as joint attention and play (Kasari et al., 2012). These interventions focus on basic skills, such as functional communication, imitation, and basic receptive and expressive language learning skills (Pelios et al., 2004).

### Technology-based intervention in autism spectrum disorder

Assistive technology or technology-based intervention refers to the use of an electronic or digital device, application, or software that helps improve a specific skill (Syriopoulou-Delli and Gkiolnta, 2020). The implementation of technology-based interventions in the field of the education of atypical pupils is considered an increasing trend in many countries. This intervention has gained recognition among teachers, parents, and practitioners (Qahmash, 2018). The technology-based intervention seeks to train many skills, such as social communication, face recognition, academic skills, vocabulary (Massaro and Bosseler, 2006), and communication skills (Gevarter et al., 2020).

Many researchers and clinicians have noted the benefits and advantages of technology-based intervention specifically for people with ASD (Grynszpan et al., 2014) for various reasons.

Firstly, technologies such as mobile phones and tablets are relatively affordable and socially valued (Light and McNaughton, 2012). Second, mobile phone and computer-based interventions (CBI), also known in the literature as computer-assisted intervention (CAI) or computer-assisted learning (CAL), can assist children in expanding their attention span and increasing motivation (Novack et al., 2019). These interventions can also aid in automated practice and feedback, and can be easily programmed. Another valuable component is the potential to present multiple sources of information, such as text, sound, and images, in parallel. Massaro and Boessler (2003) tested this in a study in which all students showed an increase in identification accuracy once training was implemented. Additionally, the children generalized the learned vocabulary to new instances of vocabulary items. Third, tablets can be attractive for young learners, providing opportunities for self-initiation or prompting the child with few stimuli (Stockall and Dennis, 2014). Fourth, mobile technologies could increase interaction and participation within a learning environment and, more importantly, facilitate the learning process (Qahmash, 2018). Fifth, students with ASD learn from visual media and pictures are one of the first supports used to acquire language. Technology makes visual images more accessible to students with ASD (Odunukwe, 2019). Despite these alleged advantages, empirical evidence on whether technology-based intervention for vocabulary learning is more beneficial than using methods without the use of technology has not been reviewed systematically and therefore an overall perspective of the state of current research is lacking (Goldsmith and Leblanc, 2004).

### Previous reviews

Over the past 10 years, some systematic reviews and studies have contributed to achieving a picture of the value of the different technological methods used for the intervention to help children with ASD improve different abilities. Previous systematic reviews have evaluated technologies used as intervention tools for language and literacy, social skills, and emotion recognition with technology-based interventions.

The first systematic review was conducted by Ramdoss et al. (2011). The authors evaluated CBI to teach communication skills to children with ASD in studies from 1990. The systematic review produced 10 studies. However, due to the variety of literacy skills targeted for instruction in different studies and the heterogeneity of participants, according to the authors, it was not possible to draw a conclusion regarding the effectiveness of CBI in teaching literacy skills to students with ASD.

Another systematic review on this type of intervention in the autism population is the one carried out by Fletcher-Watson (2014). The aim of the review was to identify common characteristics of different interventions and to review a consistent research model using technology. The information was extracted on how technologies were designed, implemented and evaluated to define best practices. Fletcher-Watson (2014) collected evidence over more than four decades of research. The search was conducted in 2011 and 2013, and a final list of 52 studies was reviewed. The CBI approach appeared to show an advantage over traditional teaching methods in academic learning, social skills, and life skills development.

Later, Grynszpan et al. (2014) conducted another systematic review and meta-analysis to assess innovative technology interventions for children with ASD. These authors evaluated the efficacy of studies using pre-post-intervention designs between January 1990 and December 2011. Twenty-two articles were found. Their results demonstrated an overall significant effect size for the controlled studies and a similar effect size for the randomized control studies. According to Grynszpan et al. (2014), the significant effect size might support the efficacy of innovative technology. However, the authors pointed out some differences between the studies, such as the characteristics of the participants, the procedure, and the methodological approaches.

Another systematic review, by Aljameel et al. (2018), reviewed different technologies and different contexts and evaluation methods that were used to improve emotion recognition, social skills, and language skills for children with ASD aged 10–16. Nineteen articles were reviewed (from 2005 to the end of 2015). The results indicated that the children showed sufficient progress in learning within the CBI paradigm. However, according to the authors, future research must demonstrate the effectiveness of technologies by using a larger number of participants and indicating differences in functional abilities of children diagnosed with ASD.

The systematic review carried out by Valencia et al. (2019) evaluated how the use of technology contributes to the education of people with ASD, what user experience and accessibility elements or methods were considered when analyzing the impact of technology on people with ASD, and what game elements were considered when using gamification or serious games in education. They examined 94 studies published between January 2009 and June 2019 focusing on those conducted in an educational context or focused on teaching. The results showed that technology was useful in promoting constant learning for people with ASD.

Lastly, the meta-analysis conducted by Sandgreen et al. (2021) to review digital interventions in the treatment of people with ASD of any age found 19 articles (prior to June 2019). The review presents the different technological devices used, the skills targeted, and the effect size of interventions, and concludes that computer programs constitute the technological solution most frequently used, the skills targeted, social skills, and effect size was positive, however, small. This study faced challenges in drawing conclusions due to significant variations in effect sizes and concerns about the risk of bias. In conclusion, this systematic review and meta-analysis considers that the digital intervention for people with ASD is currently too heterogeneous, making comparison with other approaches difficult.

To our knowledge, although these systematic reviews provide extensive overviews of the field, none of the systematic reviews specifically assess vocabulary learning. Previous meta-analyses and systematic reviews have evaluated only the use of the digital intervention to assess other skills for people with ASD (Sandgreen et al., 2021).

The present review aims to (a) assess the level of evidence in studies aimed at evaluating the effectiveness of technology-based interventions used in children and adolescents with ASD, specifically focusing on interventions that target receptive and expressive vocabulary acquisition. Additionally, it aims to (b) review the conclusions of studies which set out to compare the effectiveness of technology-based intervention methods with teaching methods without technology in this same population.

## Methods

The present review uses a narrative approach, conducted to provide an overview of studies that involved the use of digital devices (that is, CBI, robots, and tablets) that focused on expressive and receptive vocabulary interventions for children with ASD. This protocol was registered with the PROSPERO International Prospective Register of Systematic Reviews (CRD42021238758).

### Study characteristics

Eligible studies met the following criteria: participants with ASD, published in English during the period of 2006–2022, participants between 0 and 16 years of age, and intervention using technology that has been designed to improve vocabulary skills (i.e., using any type of technological device for the intervention, such as tablets, computers, robots, etc.). Studies that used technology to improve oral communication that do not specifically include vocabulary were excluded.

Interventions with children with ASD included in this review could be performed by any practitioner and directly by specialists themselves or through parents, teachers, or teaching assistants. Additionally, these interventions were implemented in various settings, such as homes, schools, clinics, or private practices.

### Comparison groups

Studies with and without a comparison group were included. Control groups included no treatment, treatment as usual, or other treatment, with or without digital technology. Children subjected to intervention (or experimental group) included those receiving therapy through any digital device. Both comparison groups involved children with ASD.

### Information sources

The databases used to obtain studies for this review were: Education Resources Information Centre (ERIC), MEDLINE, PsycINFO, PubMed, SCOPUS, and Web of Science (WOS). The search strategy was first developed in WOS and then adapted to the other databases. Keyword fields in all five databases were searched using Boolean terms (Autis* OR Asperger OR ASD) AND (Intervention OR instruction OR teaching OR therapy OR training OR treatment OR learn*) AND (language OR vocabulary OR literacy OR lexicon OR communication) AND (technology OR machine OR ‘computer assisted’ OR computer assisted OR multimedia OR digital OR ‘robot assisted’ OR robot assisted).

### Study selection and data extraction

**Figure 1:**
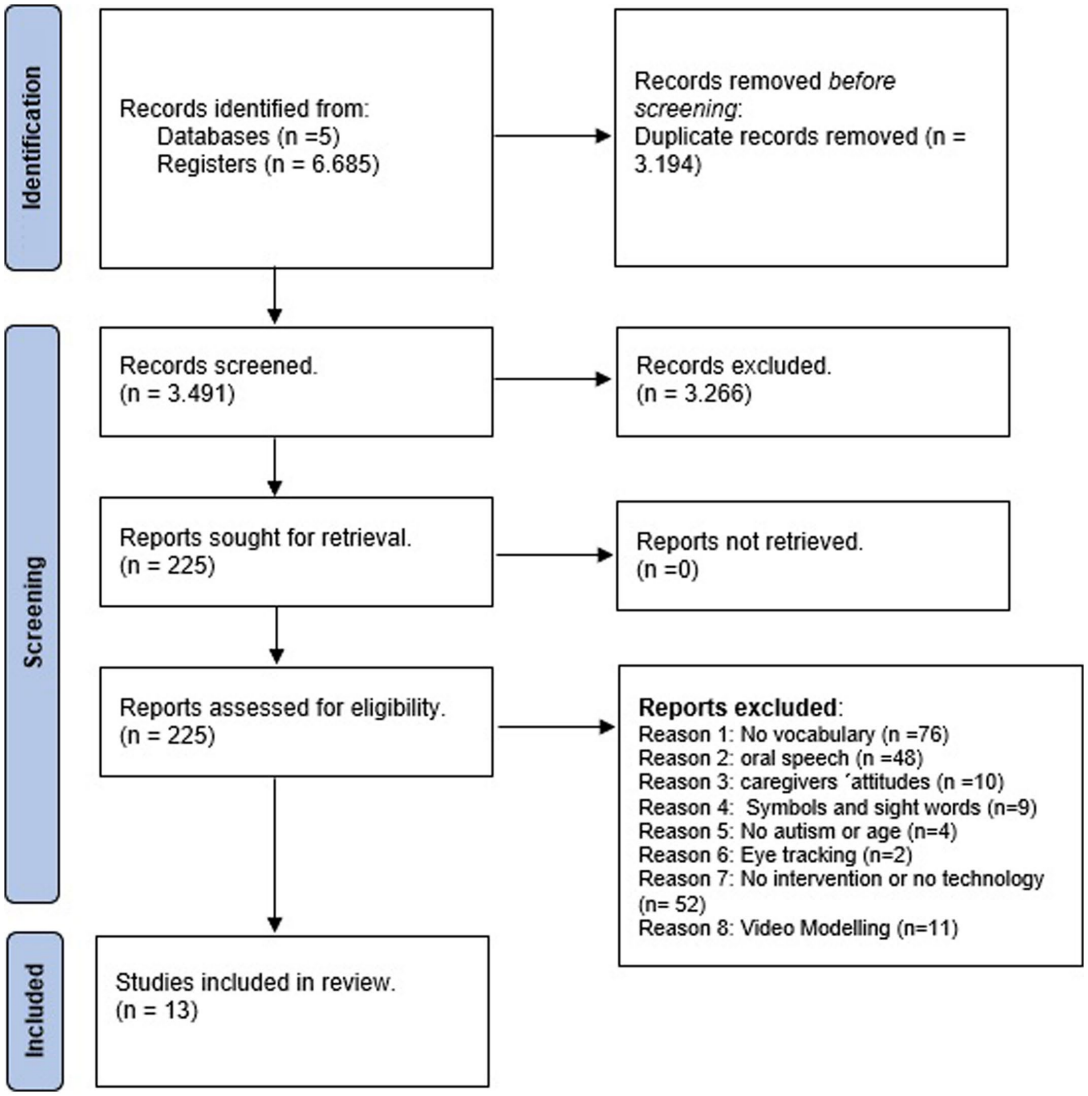
The systematic process of identification, screening, eligibility, and inclusion of the studies. The diagram is an adapted version of the PRISMA flow diagram (Page et al., 2021).

The study selection was carried out in three stages by one of the authors and the precision was verified by another. For this review, the study selection and screening adhered to the guidelines of Preferred Reporting Items for Systematic Reviews and Meta-Analysis (PRISMA) (Figure 1).

Stage 1: Searches were conducted in March–April 2021 and updated in September 2022 using the six databases. For further screening, all citations were exported to Rayyan software (Ouzzani et al., 2016) which was also utilized to remove duplicates.

Stage 2: After Stage 1, all papers underwent screening based on title and abstract. Two independent reviewers double screened all articles according to inclusion and exclusion criteria, as well as intervention information. An overall agreement of 80% was achieved before resolving the discrepancies.

Stage 3: Articles obtained in the previous stage underwent a second round of screening by two independent reviewers based on full text. This stage aimed to include articles specifically focused on: (a) vocabulary learning, (b) population with ASD, and (c) technology-based intervention. Overall agreement was reached through consensus between the two independent reviewers.

### Data extraction

From studies that met the inclusion criteria, we extracted data on participant characteristics, diagnosis, research design, type of intervention used, settings, software used, language, diagnostic measures, main outcomes, and secondary outcomes.

### Methodological quality

We evaluated methodological quality using the Sterne et al. (2019). Five characteristics were used for the evaluation: randomization process, evaluation of the effects of the intervention, missing outcome data, measurement of the outcome and selection of the reported results (low, medium, or high risk). For single-case designs, Kratochwill et al. (2010) criteria (WWC) were used: (i) the independent variable must be systematically manipulated, (ii) the outcome variable must be measured systematically, and (iii) the study must include at least three attempts to demonstrate an intervention effect. Based on the results of each of these criteria, each study was classified as *Meets without Reservation, Meets with Reservations, or does not meet WWC Single-Case Design Standards*. The evaluation was carried out by the same two authors and the results were discussed until agreement was reached.

## Results

To answer the research questions of this review, the studies were divided into two groups. The first group refers to studies that explored the efficacy of technology-based intervention itself (without comparison), and the second group refers to studies that compared technology-based intervention and nontechnology-based interventions. The studies we found in the second group only compared gain scores or were designed to measure time to success. Their analyses or designs, therefore, did not allow them to be added to the first group, and thus both categories of studies were mutually exclusive.

### Study selection

After applying each of the filters described in the study selection criteria, a total of 13 studies published were obtained within the period of 2006–2022 were obtained. Figure 1 includes the systematic process for identification, screening, eligibility, and inclusion of the studies using the adapted version of the PRISMA flow diagram (Page et al., 2021). The excluded studies did not provide a vocabulary intervention (*n* = 76), the objective was to acquire oral language in general without vocabulary measures (*n* = 48), they only taught symbols and sight words (*n* = 9), they did not describe an intervention (*n* = 52), only evaluated the attitude or perspective of caregivers concerning technology-based intervention (*n* = 10), the technology used was eye tracking (*n* = 2), the intervention included video modeling (*n* = 11), or lastly, the age and diagnosis did not match with the inclusion criteria of this systematic review (*n* = 4).

### Characteristics of the studies

Of the 13 studies evaluated, nine used a single-case design (Mulholland et al., 2008; Kagohara et al., 2010; Ganz et al., 2014, 2015; Chebli et al., 2017, 2019; McKissick et al., 2018; Khowaja and Salim, 2019; Pellegrino et al., 2020), and four were group studies (Whalen et al., 2010; Allen et al., 2015; Novack et al., 2019; Pellecchia et al., 2020). The ages of the participants in the different interventions are found in Table 1. The duration of the vocabulary intervention is presented in Table 2. The duration of the different studies varies: nine studies held the sessions 3–5 days a week, one study 3 h for 4 weeks, one study 4 sessions one per week, and two studies did not provide information about duration.

**Table 1:** Key elements of the studies reviewed.

Author single-case studies	Number of participants; ages in years	Gender (M, F)	Aim of the study	Technological device	Instructional content	Main results
Ganz et al. (2014)	3, Ages: 8,9,14	Both (2: M, 1: F)	Investigates the effects of a visual script delivered using the iPad on the use of verbs and nouns.	*Tablet*	Treatment Condition: The same for baseline and intervention. Baseline: The researcher showed a 45 s video and asked the participants “what is happening?,” and no prompts or cues were given. Intervention: Tablet was turned on and placed next to the child a 45 s. video was shown to participants followed by researchers’ question “what is happening.” The child had 10 s to point out the correct answer. Nontreatment condition: Same as for treatment but the iPad was turned off.	Two of the three participants demonstrated significant increases in the use of nouns and verbs. The participants demonstrated a generalization of the use of nouns and verbs provided by parents or teachers.
Chebli et al. (2019)	7, age: 5–9 years	Both (5: M, 2: F)	Compare the effectiveness of tablet- delivered to instructor-delivered teaching and evaluate generalization of concepts taught to 3-dimensional representations. Assess maintenance of correct response and compare nonresponding across modalities	*Tablet*	Baseline and teaching on tablet condition: 3 Images were presented on screen. Generalization: Instructor presented 5 real objects. A digital voice named the word, and the child was required to choose the image associated with the concept by selecting it on the screen. Baseline and teaching on instructor condition: Instructor presented 3 images on paper. Generalization: Instructor presented 5 real objects.	Five participants showed better maintenance learnt with the instructor, and the results of two participants were the same in both conditions. Nine out of fourteen concepts were generalized more rapidly after the instructor delivered them. Five participants showed better maintenance of the concepts learnt with the instructor and, for two participants, the same across modalities. Six out of seven participants showed lower levels of nonresponding during instructor-delivered and only 1 less nonresponding with the tablet condition.
Chebli et al. (2017)	5, age: 4–11 years	Both (2:M, 3: F)	Extend research on teaching one-word concepts by evaluating generalization to pictures and objects while minimizing trainer involvement to individuals with ASD.	*Tablet*	Baseline: Images were presented on the tablet with one image depicting the target concept and two distractors. The automated voice named the concept, and the child had to choose the image associated with the concepts by selecting it on screen. Training: Like the baseline session with prompts. No social reinforcement was used; only the preferred video was automatically used by the app. Generalization: Conducted by an experimenter and real objects.	Three out of five participants generalized on at least two concepts following tablet-based instructions. By integrating video reinforcement within the app to promote learnt independence, minimized trainer involvement. Two children were able to maintain correct response to these concepts for several weeks after training. Two of 5 children never showed generalization. After tablet-assisted condition.
Ganz et al. (2015)	1, age: 4	(1:M)	Determine whether the use of PECS-based communication instruction improves the receptive language identification of target words of one child with autism.	*Tablet*	Baseline: PECs app was muted. Objects and icons not verbally labeled. The icons in the PECS app were presented and the child had to choose the concept by touching the screen. Instruction phase: The sound was turned on.	No convincing evidence of a strong, clear functional relation between SGD intervention and receptive identification. The intervention appears to improve the responses levels for two of the target words. The participants did not make a connection between the spoken word and the image presented.
Kagohara et al. (2012), Study 2	2, age 13 and 17 (17-year-old data not considered)	(2:M)	Expand the participants vocabulary by teaching a set of 18 drawings presented in a commonly used picture book and using an iPad as the SGD.	*Tablet*	The pages with color line drawings when touched produced the corresponding speech output. Baseline: iPad and book placed on the table in front of the participant. The trainer pointed to one drawing and asked, “what is this?” Intervention: The same as in the baseline except that prompting was used if no correct answer within 10 s.	The two participants did not have correct responses during baseline. However, with the intervention, the performance increased above 85 and 100%. Acquiring a new and larger set of picture naming responses.
Pellegrino et al. (2020)	3, age: 4, 5, 5	(3:M)	Evaluate to what extent stimuli delivered via tablet versus flashcard increased and maintained receptive labeling in young children with ASD. In addition, participant preferences for stimuli delivery were assessed.	*Tablet*	Pre experiment: Stimuli presented by flashcards with the instruction “touch (stimulus). Teaching session: The general procedure was identical under all conditions; 3 stimuli were presented to teach receptive labeling in a discrete trial instruction format. Each of the 3 stimuli was presented 5 times in random order. Flashcard condition: The stimuli were on laminated cards; instructor manually rotated the order of the cards. Tablet condition: The stimuli were presented on a tablet; the instructor manually rotated the order of the stimuli.	Participants showed a preference to work under tablet conditions. One participant demonstrated preference for flashcards after 10 sessions. And the other two participants demonstrated preference for the tablet after 10 and 5 sessions. All participants demonstrated approximately equivalent performance in maintenance across conditions.
McKissick et al. (2018)	3, age: 14 and 13	Both (2:M,1: F)	Effect of CAI on the acquisition of grade-aligned science vocabulary. What are the students’ and teachers’ opinions on using the CAI package used to teach science vocabulary?	*Computer*	Probe slideshow: Consisted of 10 total slides. Five slides showed a picture of an amoeba and asked them to identify the target structure on a picture (what is the arrow pointing to?). Five slides asked them to fill in a statement regarding the function of the target structure. CAI intervention slideshow: Consisted of 31 slides. Included explicit instruction and video slides. CAI consisted of 31 slides. Including explicit instruction and video slides.	The three participants, the classroom teacher, and paraprofessionals agreed that the intervention was effective in teaching the targeted skills.
Novack et al. (2019)	28, Age: 3–8	Both (24:M, 4: F)	Investigate the effectiveness of Camp Discovery in teaching receptive language skills.	*Tablet*	Camp Discovery a mobile application incorporated modifies Discrete-Trial Training DTT, in which user is asked to identify a specific target with variations of instruction and variety of lessons.	Participants made a significant gain of a course of 4 weeks and maintained the acquires skills following 1 month.
Khowaja and Salim (2019)	5, Age: 6–10 years	(5:M)	Examine the effectiveness of the prototype SG “vocab builder” to improve the performance of learning vocabulary among children with autism.	*Computer*	Baseline: Questions are asked to identify the correct image of an item by showing three images, conducted to measure the current level of knowledge. Intervention: Each session corresponds to learning of one vocabulary item; once the participant saw and memorized all images associated with the item, they were asked to identify the current image of the item. Each day player played an activity game for 3 min.	The number of correct responses to receptively identify vocabulary items among children with ASD improved from baseline to intervention and was maintained at the end of week 1 and 2 after the withdrawal of the intervention. This shows that the prototype was effective in facilitating children with ASD to learn vocabulary.
Mulholland et al. (2008)	3, Age: 5–9 years	(3:M)	Effectiveness of teaming up with the Timo app to support expressive and receptive language development.	*Computer*	Virtual tutor called Timo. The tutor highlighted a picture and told the student its name. The program randomly moved the pictures around the screen and the tutor asked the student to click on the picture reflecting the word. As the student progressed through the lesson, the participant was asked to identify pictures by clicking on the one spoken by the tutor.	Three of the five students demonstrated improvement in language skills. It is not clear which of the participants who demonstrated improvement have autism.
Author group study	Number of participants; ages in years	Gender (M, F)	Aim of the study	Technological device	Instructional content	Main results
Pellecchia et al. (2020)	154, Ages: 5 to 9		The effectiveness of CAI designed to improve children’s expressive and receptive language, cognitive, and academic skills was evaluated.	*Computer*	Computer lessons incorporate principles of ABA using a discrete trial format in which the student is provided with a specific instruction and selects the correct response. The program professes to have 5 levels of difficulty. Offline activities: Teachers provide interpersonal lessons by direct instruction following the same areas targeted in CAI activities. And administered at the beginning and end of the school year two measures as pre and post.	Showed an overall null effect for the Teach town: Basic program between groups (offline and computer lessons). The mean change score on DAS-II and BBCS scale were not statistically different between groups
Whalen et al. (2010)	47, 3–6		Assessed the effectiveness of Teach Town: basics in a randomized trial implemented in special education program	*Computer*	TeachTown:Basic a CAI program that includes computer lessons and natural environment activities using a discrete trial format where they receive reinforcement for correct responses. TeachTown Connection off-computer activities are lessons plans to implement in the natural environment for students to work on skills that are not targeted in the computer and enhance generalization of skills learned on the computer to the natural environment	The majority students demonstrated a significant progress in the software program by mastered lessons across the four learning domains than the control group. The standardized outcome measure were shown in changes of raw scored on PPVT-III and Brigance Inventory
Allen et al. (2015)	16, 4–16	(16:M)	Children with ASD are better able to learn new word-referent relations using an iPad or traditional picture book	*Tablet*	Stimuli were color photographs presented via picture books and tablet. Book condition: book placed in front of the child and experimenter turned the pages. iPad condition: children controlled their transition between pictured in both the experimenter directed the child’s attention verbally or audio recording on iPad	The mapping test revealed that medium of presentation iPad or Book did not impact on extension of labels did not find an advantage for learning with iPad.

**Table 2:** Method classification.

Single case studies	App or program used	Duration	Instructor	Setting	
Ganz et al. (2014)	iCommunicate app	Each day for 3 days a week	Second author and occasionally first author	School and the home of the participant.	
Chebli et al. (2019)	The Open-Source Discrete Trial Instructor app- developed by the research team.	6–12 sessions per day, 3 days a week.	First author	School	
Chebli et al. (2017)	The Open-Source Discrete Trial Instructor app- developed by the research team.	4–8 sessions per day, 3 days a week.	First and second authors	School	
Ganz et al. (2015)	PECS phase III app	Not specified	First and second authors	Autism clinic	
Khowaja and Salim (2019)	SG prototype	5 weeks. 2–3 days a week	Not specified	Not specified	
Mulholland et al. (2008)	Software Team up with Timo	3 days a week	First author	Not specified	
Kagohara et al. (2012)	Book with pages with color line drawing and tablet	2–4 sessions per week. Each session lasted 15 min.	First author	School	
Pellegrino et al. (2020)	Stimuli on tablet	3–5 days a week.	Instructor- not specified	Preschool	
McKissick et al. (2018)	Slideshows	Not specified	Baseline: First and second observers, intervention: first author.	School of Special Education	
Novack et al. (2019)	Camp discovery mobile application	3 h per week for 4 weeks	Research assistant	Participant’s home or treatment center	
Group studies	App or program used	Duration	Instructor	Setting	
Pellecchia et al. (2020)	Teach town: basics	5 days per week. One year	Teacher	School	
Whalen et al. (2010)	Teach town: basics	5 days per week. Three months	Teacher	School	
Allen et al. (2015)	Stimuli on tablet and books	4 sessions for 4 weeks	Experimenter	School	

Regarding the diagnosis of autism in the 13 studies, in seven studies the diagnosis had been carried out with a specialist using different standardized diagnostic scales [e.g., Autism Spectrum Rating Scale (ASRS), Childhood Autism Rating Scale (CARS), Gilliam Autism Rating Scale (GARS)] (Table 3). Four studies did not specify the diagnostic process of the participants and two studies described the diagnosis as applied by a pediatrician or diagnostic specialist (without indication of instruments).

**Table 3:** Sample classification.

Author single- case studies	Diagnosis	Scales used to assess diagnosis	Autism symptoms as reported in studies.
Ganz et al. (2014)	Autism and speech impairment	Autism Spectrum Rating Scale ASRS (Goldstein and Naglieri, 2009) DSM-TR-IV (American Psychiatric Association, 2000)	ASRS total: Elevated DSM-IV-TR: Very elevated
Chebli et al. (2019)	Autism	DSM-TR-IV (American Psychiatric Association, 2000) Childhood Autism Ratings Scale- 2 CARS-2 (Schopler et al., 2010) Adaptive Behavior Assessment System- Second Edition (Harrison and Oakland, 2003)	N = 3: Severe symptoms N = 3: Moderate mild symptoms N = 1: Mild symptoms
Chebli et al. (2017)	Autism	DSM-TR-IV (American Psychiatric Association, 2000) Childhood autism rating scale-second edition CARS-2 (Schopler et al., 2010)	N = 3: Mild to moderate N = 1: Mild symptoms N = 1: Severe symptoms
Ganz et al. (2015)	Autism	Autism Spectrum Rating Scale (Goldstein and Naglieri, 2009) DSM-TR-IV (American Psychiatric Association, 2000)	ASRS: Elevated DSM-IV-TR: Elevated
Pellegrino et al. (2020)	Autism	Diagnosis by a pediatrician or diagnostic specialist. Verbal Behavior Milestones Assessment and Placement Program	N = 1: Level 3 N = 2: Level 2
McKissick et al. (2018)	Autism	GARS Gilliam Autism Rating Scale (Gilliam, 1995)	N = 1: Require support N = 1: Require substantial support N = 1: Not specified
Khowaja and Salim (2019)	Autism	Not specified	Require support
Mulholland et al. (2008)	Autism	Not specified	Not specified
Kagohara et al. (2012)	Autism and severe intellectual disability; autism and obsessive-compulsive disorder	Vineland adaptive behaviors scales Vineland-II (Sparrow et al., 2005)	Not specified
Novack et al. (2019)	Autism	Vineland adaptive behaviors scales Vineland-II (Sparrow et al., 2005)	Not specified
Author group study	Diagnosis	Scales used to assess diagnosis	Autism symptoms as reported in studies
Pellecchia et al. (2020)	Autism	Autism Diagnostic Observation Scale, Second Edition (ADOS-2)	Not specified
Whalen et al. (2010)	Autism	CARS-2 (Schopler et al., 2010) Adaptive Behavior Assessment System-Second Edition (Harrison and Oakland, 2003)	Severely autistic
Allen et al. (2015)	Autism	Diagnosis from a qualified educational or clinical psychologist	Not specified

(N) Number of participants. DSM-TR-IV: Diagnostic and Statistical Manual of Mental Disorders.

Regarding the technological devices used to teach vocabulary, their variety was very limited and consisted only of computers and tablets.

Five studies used computers (Whalen et al., 2010; Pellecchia et al., 2020) used a software called TeachTown Basic for implementing CBI; Khowaja and Salim (2019) also included computer to teach vocabulary by listening to verbal instruction given by the computer; McKissick et al. (2018) used explicit and video-based instructional slides in the computer to teach vocabulary, and Mulholland et al. (2008) used a software named Team Up with Timo as a tutor.

Eight studies used a tablet device to teach vocabulary. Three studies used an Android device and five iOS devices. The use of the tablet to teach vocabulary varied among studies: Two used an app designed by the research team, three showed images to teach vocabulary, one used a PECS app, one used an app called Camp Discovery app, and the last one used the iCommunicate app.

### Efficacy of technology-based interventions

Eight studies explored the use of technology-based interventions to teach vocabulary for children with ASD without a comparison to non-technological interventions.

Ganz et al. (2014) conducted a treatment study with a sample of three, 8–14 years old, a participant with very elevated levels of autism symptoms according to DSM-IV. In all three participants, social communication was characterized by difficulty in initiating and maintaining relationships and emotional regulation, and all three showed unusual behaviors that included repetitive speech and motor movements. Two students were able to imitate multiple-word phrases, used spontaneous speech rarely, and had difficulties to understand, the third participant spoke in three-word or longer phrases, and sometimes used spontaneous speech. In this study, participants had to answer what happened in a 45-s video using a tablet. The tablet was turned on and placed next to the child on the table. Least-to-most prompting was used when the students did not correctly answer the question “What is happening.” The results were mixed, as two of the three participants who used spontaneous speech demonstrated a significant increase in the use of nouns and verbs, and the same two participants required less intrusive prompts over time, while the prompts of the third participants were consistent throughout the study. All three demonstrated a generalization of the use of nouns and verbs provided by a parent or teacher rather than by researchers.

Another study by Ganz et al. (2015) examined whether the use of a tablet with PECS-based communication instruction improved the receptive-language identification of target words. In this case, the tablet was used as a Speech Generating Device (SGD), that is, the tablet allows children to select a picture, symbol, letter, or word and reproduce it with voice. The study included only one 4-year-old participant, who had severe language disorders and an elevated level of autism according to the CASRS scale (Schopler et al., 2010). The participant communicated with single words, made eye contact, responded to simple demands with a gestural prompt, and had limited language skills. The multiple-baseline study consisted of different phases. In the first phase, the child had to touch an icon to reach an object using the PECS app; in this phase, the sound was muted and the objects and icons were not verbally labeled. In the second phase, the instruction phase, five icons on a PECS app were presented to the participant, sound was turned on, and if the participant touched an icon, it produced a recorded voice, then the researcher enticed the participant with two objects; if the participant selected an icon on the tablet, the researcher said “Take it.” If the participant did not reach for the object that did not correspond to the icon, a four-step error-correction procedure was performed. Although there was no convincing evidence of a clear relation between the SGD intervention and receptive identification, the participant improved the response levels for two out of three target words and showed a higher mean level and an increasing trend from baseline to intervention. However, the participant was unable to make a connection between the spoken word and the image.

The study by Kagohara et al. (2012) used a tablet as a SGD to teach words for 18 colored drawings from a commonly used picture vocabulary book for children. Two adolescents of 13 and 17 years of age participated in this study. They had an expressive language of less than 2.5 years, as determined by the results of the Vineland Adaptive Behavior Scales. According to the author, both participants rarely spoke and the speech was mainly inaudible without specific details of the described symptoms of autism. A tablet and a book were placed in front of each participant. The trainer asked a question while pointing at one line drawing from the book and asked the participant to use the tablet to give the correct answer. During the baseline, neither of the students made any correct responses; however, with intervention, their performance increased. Therefore, the multiple-probe design showed that the implementation of this type of instructional procedure increased the correct picture-naming responses and that the two participants had acquired a new set of picture-naming responses. But the follow-up sessions were relatively short and lacked generalization probes.

Chebli et al. (2017) used a tablet device to evaluate the effects of tablet-based instruction and generalization in five 4- to 11-year-old children with ASD. An automated voice named the concept, and the child had to choose the image associated with the concept by touching the screen; the only reinforcer was a preferred video. The generalization was evaluated with five different untaught examples of the target concept. Two participants had mild to moderate ASD symptoms in CARS-2 and had no formal means of communication. A third participant had mild symptoms of ASD and spoke four- to five-word sentences with unclear pronunciation. The fourth participant had mild to moderate symptoms of ASD and the fifth participant had severe symptoms of ASD, both using one-word concepts to make simple requests. The authors of this study stated mixed results; only three of the five participants generalized in at least two concepts after tablet-assisted instruction, three of them had mild to moderate symptoms of ASD. The participant with severe ASD symptoms had the highest levels of nonresponses and required a greater number of prompts to do the task.

Novack et al. (2019) conducted a randomized controlled design with 28 participants aged 3–8 years. All participants were verbal. The purpose of this study was to investigate the effectiveness of Camp Discovery in teaching receptive language skills across a variety of domains to children with ASD. Camp Discovery is a mobile application that incorporates modified Discrete-Trial Training DTT procedures and other behavioral principals of ABA. To initiate treatment sessions, the researcher opened the tablet with Camp Discovery and instructed the participant to select one of the target lessons. Participants were randomly assigned to the immediate treatment (IT) group or the delayed treatment control (DTC) group; both benefited from the application. The IT group began interacting after the initial probe, whereas the DTC continued with treatment as usual with no manipulations; after 4 weeks both groups received a second probe. Following the second probe, the DTC group appeared to benefit from the treatment phase. Participants learned significantly more in the IT group than the DTC group. Participants made a significant gain in gameplay over the course of 4 weeks.

Khowaja and Salim (2019) evaluated the learning environment of a game to teach bird vocabulary in five children aged 6 to 10 years of age with ASD while listening to a computer-provided verbal instruction. All participants had ASD with required level of support, had difficulty learning different categories of vocabulary, and had a basic knowledge of computers. The participants had to identify the correct word by selecting one of three images. They improved on bird learning after using the prototype and retained the names of birds after the first and second weeks after the intervention was over.

McKissick et al. (2018) evaluated the effect of CAI for the acquisition of science vocabulary in middle school students with ASD. The participants were three middle school students aged 13 and 14 years. Two students used functional speech to communicate all desires and needs, and one student relied on verbal communication. The CAI intervention consisted of slides which included both explicit instructions and videos edited to highlight the explanation of the function of the cell membrane. Following the video, a slide appeared showing the amoeba with an arrow pointing to the cell membrane, and after a model slide, the question for the participant appeared with four options to answer. During baseline conditions, all three participants had low levels of correct responses, but all demonstrated improved performance over time. There appeared to be a functional relation between the CAI intervention and an increase in the number of correct response items answered correctly during the sessions.

Lastly, Mulholland et al. (2008) showed the effectiveness of using an animated software program called Team Up with Timo to teach expressive and receptive language by providing audiovisual animations for three participants with ASD, ages 5–9. The animated tutor was programmed according to each child’s name and words to learn. The tutor highlighted a picture and told the student its name, and then the tutor randomly asked the student to click on the picture that reflecting the word the tutor used. Some students were asked to identify the pictures by clicking on the word spoken by the tutor, and others were asked to name the pictures as the tutor highlighted them. In the study, three of the five students demonstrated improved language skills in the post-test results. However, it is not clear which of those five participants were the three with ASD.

Overall, three of the eight studies described above used CAI, while five used tablets as a tool for vocabulary intervention. Interestingly, the three studies that used CAI showed positive results. With these results, it is not possible to draw a conclusion regarding the effectiveness of CAI in children with ASD, as the procedure, sample, and participants characteristics are too heterogeneous. However, in the studies that used a tablet as a tool, in four of the five studies the results are mixed, suggesting that while using a tablet may benefit some students in generalizing language concepts, for others it may be a less useful tool.

### Technology vs. nontechnology-based intervention

Three group (Whalen et al., 2010; Allen et al., 2015; Pellecchia et al., 2020) and two single-case studies (Chebli et al., 2019; Pellegrino et al., 2020) compared technology-based and nontechnology-based instruction, thus providing information about the added value of technology.

Whalen et al. (2010) and Pellecchia et al. (2020) used a CAI intervention with software called TeachTown, designed to increase students’ vocabulary and listening skills. The program included different ABA lessons, in which the program gave an instruction and participants had to select the correct response that had different levels of difficulty.

In Whalen et al. (2010), the TeachTown connection off-computer activities were lessons conducted in natural environments for students to work on skills not addressed on the computer and to enhance the generalization of skills learned on the computer to natural settings. Forty-seven participants joined from preschool and kindergarten/first grade classes, all identified as students with severe autism. Children with ASD aged 3–6, who had received TeachTown: Basic for 3 months, showed greater gains in all domain lessons, with 15 out of the 22 treatment group students demonstrating significant improvement. In the second study (Pellecchia et al., 2020), for the control group, teachers delivered lessons that targeted the same areas as the program. One hundred fifty-four participants joined from kindergarten through second grade autism support classrooms; however, specific symptoms of autism were not specified. Eighty-four children with ASD aged 5–9 years in the treatment group, who had received TeachTown for one academic year, did not show greater gains in receptive or expressive language compared to children in the control group (those without technology).

In a design within-subject, Allen et al. (2015) compared whether children with ASD learned new word-referent relationships better using an iPad or a traditional picture book. Sixteen male children with ASD, aged 4 to 16 years, participated in this study. Stimuli consisted of color photographs presented through either picture books or an Apple iPad 2. In the book condition, the experimenter pointed to each training picture and verbally directed the child’s attention. In the iPad condition, the iPad was placed in front of the child, allowing them to control their transition between pictures. The results did not indicate an advantage for learning with the iPad; the medium of presentation, whether iPad or book, did not affect the extension of labels by children.

A single-case study (Chebli et al., 2019) compared technology versus non-technology using a tablet as a digital device. The author recruited seven children with ASD from 5 to 9 years of age. Two participants had severe symptoms of ASD and did not have means of communication. Other three participants had mild to moderate ASD symptoms and used one word to communicate, while participant number six had mild to moderate ASD symptoms and meaningful sentences of three-to-five-word sentences, and, lastly, participant number seven had severe ASD symptoms and sometimes used a word statement with unclear pronunciation. In this study, the authors evaluated the generalization of concepts. In the tablet condition, the authors presented three images, one as a target concept, and two distractors. An automated digital voice named the concept and the child chose the associated image. In the non-tablet condition, the instructor presented three images on paper. As reinforcement, the tablet played a preferred small video, the reinforcement for some participants was different, some watched videos, and some participants received their preferred food. The generalization in both conditions was conducted by the instructor. The instructor named the target, and the child had to select the item. Two participants who had severe symptoms of ASD showed a faster generalization after instructor-led teaching. The remaining five participants had mixed results: two participants with mild ASD symptoms met the generalization criterion following instructor-delivered teaching on two of the three concepts and one concept using the tablet. Similarly, another participant with mild symptoms of ASD revealed a more rapid generalization after instructor-directed teaching of the first two concepts. A participant with severe ASD symptoms generalized the first two concepts with the instructor after fewer sessions. Lastly, a child with mild to moderate symptoms of ASD showed a more rapid generalization after instructor-directed teaching with the first concept and tablet condition with the second concept. In sum, nine of the 14 concepts were generalized more rapidly following instructor-condition and the remaining five concepts more rapidly using tablet condition, and almost all children engaged in less non- responding with the instructor than with the tablet.

Another study compared technology vs. non-technology (Pellegrino et al., 2020) using a flashcard and a tablet condition. In both conditions, the instructor manually rotated the order of stimuli. The procedure was the same for both conditions. The participants in this study were three boys, aged 4–5 years, who had a diagnosis of ASD. All of them had previous experience using a tablet for leisure purposes at home and school. A participant demonstrated skills at Level 3 according to the verbal behavior milestones assessment and placement program (Sundberg, 2008) and the other two participants were within Level 2. The Level 3 participant met the criterion after using a flashcard condition in one session faster compared to the tablet condition. The participant with Level 2 of verbal behavior milestones required four more sessions to reach the criterion in the tablet condition. The last participant required nine more sessions to reach the criterion in the tablet condition than in the flashcard condition. Interestingly, participants showed a preference to work with a tablet, although they required additional sessions to learn vocabulary compared to the instructor’s condition.

Overall, five studies conducted comparisons between technology and nontechnology approaches. Among them, three used a tablet device as a tool, while two used computer-assisted instruction (CAI). Regarding the effectiveness of the studies evaluating a specific computer program compared to a control group, one yielded negative results while the other yielded positive results. Among the three studies that used tablets as tools, two followed a single-case study design and reported mixed results, while one followed a within-subject design and reported no impact on word learning using either an iPad or a book.

### Quality assessment

The methodological quality assessment for the group studies was examined using the Sterne et al. (2019). Various group studies (Whalen et al., 2010; Novack et al., 2019; Pellecchia et al., 2020) showed some concerns of risk of bias, since the process of missing outcome data was not entirely clear. In another study, it was not clear whether participants were aware of the assigned intervention design (Allen et al., 2015). However, the randomization process, counterbalance, and multiple eligible analyzes of the data criteria were fully met. For single case studies, What Works Clearinghouse criteria were used. Most studies met standards without reservations (Kagohara et al., 2012; Ganz et al., 2013, 2015; Chebli et al., 2017, 2019; Khowaja and Salim, 2019; Pellegrino et al., 2020). The study by Mulholland et al. (2008) met the standards with reservations, as it did not provide detailed information about the design and did not appear to achieve interrater reliability.

## Discussion

This systematic review examines the evidence in the literature on the effectiveness of digital interventions in vocabulary for children with ASD, by exploring technology-based interventions and, when possible, comparing them with non- technology interventions. A total of 13 studies were obtained. Eight studies only evaluated the efficacy of technology without comparing with a non-technological control group, and five compared technology with non-technology.

### Efficacy of technology-based interventions

The first research question addressed the efficacy of technology-based interventions in improving vocabulary learning in children with ASD. Eight studies were reviewed, with five using tablets (Kagohara et al., 2012; Ganz et al., 2014, 2015; Chebli et al., 2017; Novack et al., 2019) and three others using computers (Mulholland et al., 2008; McKissick et al., 2018; Khowaja and Salim, 2019).

Most tablet-based studies reported positive impacts on vocabulary. For example, Kagohara et al. (2012) found that their two participants improved their picture-naming. The severity of ASD in the participants was not specified, but they presented other comorbidities (severe intellectual disability, obsessive-compulsive disorder, and attention deficit hyperactivity disorder, respectively). Similarly, in the study by Chebli et al. (2017), the effectiveness of using a tablet was demonstrated as a method to teach vocabulary in an intervention addressed to five children aged 4–11 years, all with mild-to-moderate symptoms of ASD, except the older participant who had severe symptoms of ASD. In this case, the outcomes varied: three participants (those with mild ASD symptoms) showed generalization of concepts, but the two participants who required more prompts to sit down and continue working never displayed generalization and even showed high levels of nonresponses. This, according to the authors, may indicate a lack of interest in tablet-based instruction and consequently fewer opportunities to learn the new words. Ganz et al. (2014) found positive effects of using a tablet to teach vocabulary. In their small sample size of three participants (aged 8–14 years), all with ASD and a secondary diagnosis of speech impairment, the two youngest showed an increase in correct responses in the use of nouns and verbs using fewer prompts over time. Interestingly, all three participants demonstrated generalization when provided by a parent or teacher rather than the researcher, which could mean that the participants felt more engaged with the people they often work with. Another positive outcome was found by Novack et al. (2019). Their study included 28 participants with diagnosis of Autistic Disorder, Pervasive developmental disorder not otherwise specified, and ASD. All participants were divided into the IT group and the DTC group. Participants in the IT group demonstrated significantly greater learning, as evidenced by the difference between the pre- and post-treatment scores. However, both groups experienced benefits from the application. Although the study was conducted via tablet, the author used the term CBI to designate the intervention, in the sense that it was understood as a mobile extension of a CBI application. The availability of CBI applications on mobile devices could help overcome limited access.

Ganz et al. (2015) found mixed results. Their study included only one participant, a 4-year-old boy with ASD who communicated using single words and, when given a task, sometimes closed his eyes and turned to the side. Although the participant was able to select words using PECS and often used a tablet for a different purpose, the intervention resulted in a slight improvement for two of the three vocabulary words taught, although only slightly better than chance.

Therefore, in general, tablets can be considered useful tools to teach vocabulary to children with ASD, but there are some concerns about the results obtained so far. First, in some studies (i.e., Kagohara et al., 2012) it is not possible to know if participants generalized the knowledge since no follow-up or generalization tasks were implemented. Second, in other studies, the severity of ASD is not specified and, as the study by Chebli et al. (2017) showed, those with more disruptive behaviors may benefit less from this technology and those participants with lower symptoms of ASD severity might master more words using technology according to their specific traits (Novack et al., 2019). Therefore, the presence of these behaviors could explain the absence of positive results (i.e., in the case of the only participant in the study by Ganz et al.’s study). For this reason, individualized evaluation before using tablet-based instruction appears paramount to ensure that children will benefit from it.

Another important information that must be considered when using the tablet for vocabulary intervention is the previous experience that participants may have with this device before the intervention. In two of the studies (Kagohara et al., 2012; Chebli et al., 2017), participants had previous experience with tablets, often used to provide access to reinforcement activities in their classrooms.

Finally, the context of tablet use, and more specifically the person responsible for its use, is crucial. In the study by Ganz et al. (2014), it appears that the generalization of the vocabulary learned can be facilitated when the intervention is provided by a parent or a teacher rather than the researcher.

Taking into account all of these requirements, a tablet can be a useful instrument to improve vocabulary in children with ASD under certain conditions, contributing to learner independence, and minimizing trainer involvement, which, in turn, can facilitate the implementation of interventions with multiple students simultaneously.

Regarding the use of computers for vocabulary interventions, the three studies devoted to disentangling their efficacy have found positive results. The first study is that conducted by McKissick et al. (2018), which showed a functional relationship between the CAI intervention and an increase in the number of correct responses in the learning of science vocabulary in a sample of three middle school participants with ASD and intellectual disability. Khowaja and Salim (2019) obtained another positive result in a sample of five children with ASD (within an age group of 6–10 years) who had difficulty learning vocabulary and had basic knowledge of computers. In this case, the participants improved their learning of bird names after using the prototype and retained the names of birds after the intervention (at the end of weeks 1 and 2 following the withdrawal of the intervention). Therefore, the results showed that the prototype was effective in helping children with ASD to learn vocabulary. Lastly, Mulholland et al. (2008) included a small sample of five students, three of them diagnosed with ASD. Using the software program “Team up with Timo,” they were able to teach basic vocabulary related to areas of play, food and hygiene in three of the five students. The two students who did not benefit from the animated software program were a six-year-old boy diagnosed with severe cognitive impairment who could not even use PECs, and a nine-year-old nonverbal boy who was not motivated to use the computer. However, the paper does not specify whether these students had a diagnosis of ASD.

In conclusion, the results obtained from the application of computers to teach vocabulary to children with ASD seem promising. However, the same concerns mentioned in relation to the use of tablets could be applied to computers. In the studies, the level of severity of ASD is not specifically mentioned, making it difficult to determine which children with ASD could benefit from the use of this device. However, it is apparent that the application of computers to teach vocabulary to children with ASD occurs mainly in the case of school-aged children, while tablets are applied to younger children. In addition to age, other requirements mentioned in the application of this technology include the presence of basic knowledge of computers in children to take more advantage of them (Khowaja and Salim, 2019) and the child showing motivation to work with the computer (Mulholland et al., 2008). In all cases, it is challenging to separate the benefits derived from the device itself from the particular methodology applied in each case. For example in McKissick et al. (2018) study the CAI intervention was a package with different instructional components (explicit instruction, visual aids, etc.) and therefore it was unclear which elements of the intervention caused the good results. In the case of the study by Mulholland et al. (2008), the software used allowed the teacher to personalize the animation for each student based on the needs of each student. In any case, at least the computer provided the platform on which all these useful practices could be applied and was motivating and engaging for most of the students.

It should be taken into account that the results of the studies included in this review showed a range of heterogeneity in procedure, sample, and methodologies, as observed in the systematic review by Sandgreen et al. (2021). Diverse methods and applications were used to teach vocabulary; therefore, it is challenging to draw definitive conclusions about effectiveness. Factors contributing to this variation include small sample sizes, disparities in methodologies and the use of technology-assisted intervention, and variations in the severity of autism symptoms, making generalization of results difficult. Despite these challenges, the answer to our first question is that the technology used to teach vocabulary might be effective for some participants, but is it more effective than nontechnology approaches?

### Technology vs. nontechnology-based interventions

Exploring the second research question, comparing technology-based interventions to nontechnological approaches for vocabulary development in children with ASD, five studies were reviewed: Three studies applied interventions on vocabulary using tablets, two were single-case studies (Chebli et al., 2019; Pellegrino et al., 2020) and three were group studies (Whalen et al., 2010; Allen et al., 2015; Pellecchia et al., 2020). They failed to demonstrate better outcomes for technology-assisted interventions, except Whalen et al. (2010), who showed positive gains in most of the participants.

In the single-case study by Chebli et al. (2019), the authors aimed to compare the effectiveness of tablet and instructor-delivered teaching on the receptive identification of one-word concepts. The results showed that two out of seven participants achieved a faster generalization after instructor-led instruction. The results of the remaining five participants varied across concepts, but, in general, the participants showed more rapid generalization (and lower levels of non-responding) with the instructor for most concepts taught. This result could mean that the non-technology intervention showed more effectiveness than the technology intervention, but the authors, conscientious of the great variability of their sample, preferred to conclude that some learners can benefit more from instructors while others can benefit more from tablets.

In the other single-case study by Pellegrino et al. (2020), the authors compared the learning of receptive labeling using stimuli delivered via tablet and flashcards during discrete trial instruction in three preschoolers with ASD, comparing the number of sessions required to meet a mastery criterion for label identification. They found that all participants met the criterion faster using a flashcard than under tablet condition. However, some participants preferred to use tablets instead of flashcards, although this preference was not related to overall performance during label acquisition.

Allen et al. (2015), with a sample size of 16 participants, which levels of autism were not specified, aimed to compare whether children with ASD are better able to learn new word referent relations using an iPad or a traditional picture book by using color photographs presented using the two mediums. This mapping test to learn new word referent relations showed that the medium of presentation did not have an impact on the extension of labels and therefore did not find an advantage for learning with the iPad.

To explain the advantage of instructor-led instruction over tablet-based instruction, as shown in these studies, we should consider that tablets are generally used at home and school for different purposes. Therefore, it is likely that the children were accustomed to using tablets for play rather than learning purposes. Furthermore, children could feel more comfortable with one-on-one interaction with a teacher than with a tablet (Ganz et al., 2014). But in Allen et al. (2015) children used the tablet in educational settings and reinforcement and found no differences as a result of the medium (book or iPad) used in word learning. Thus, more research is needed in which students use tablets for longer periods of time to compensate their previous experience of using them for other purposes and for different prior experience in the use of different tools for learning. For example, in the group study by Pellegrino et al. (2020), all participants knew how to use flashcards before as part of their individualized intervention, but that was not the case with tablets.

Regarding the use of computers to teach vocabulary, two group studies in this systematic review, Whalen et al. (2010) and Pellecchia et al. (2020), evaluated the effectiveness of computer-assisted instruction (CAI) by comparing it with a waitlist control group. Interestingly, in Whalen et al. (2010), 15 out of 22 children who received computer lessons for 3 months performed better across all measures than the children in the control group on standardized outcome measures. However, in Pellecchia et al. (2020), although children participated in numerous sessions throughout the year and computer lessons incorporated the principles of ABA, the CAI intervention did not show a significantly better impact on receptive or expressive language.

These studies had differences: Pellecchia et al. (2020) likely better represents most underresourced school districts compared to Whalen et al. (2010), who had better trained teachers. There were differences in participant characteristics, duration of study (1 year vs. 3 months), standardized tests used to evaluate vocabulary learning before and after tests, and offline interventions provided. In Whalen et al. (2010), interpersonal lessons using direct instruction were provided, while in Pellecchia et al. (2020), activities to implement in the natural environment and work on skills not targeted in the computer were employed, ensuring that the interpersonal area was not neglected to improve language.

In neither study were the symptoms of ASD specified; all groups were described as students with severe autism. According to Whalen et al. (2010), the students who did not master any lessons were students with severe behavioral and/or attention problems. In Pellecchia et al. (2020), the sample was not specified according to the severity of symptoms in children with ASD, making it unclear which individuals could derive greater or lesser benefits from the CAI method. In addition, concerns arise regarding the quality assessment, as the process of missing outcome data was not clear.

## Conclusion

Our systematic review examined the efficacy of technology-based interventions but found a limited number of studies. The use of a variety of technological devices was limited and consisted only of tablets and computers to teach vocabulary to children with ASD.

Tablets, in particular, demonstrated positive results in several studies, showing increased correct responses and generalization of vocabulary skills, similar to Kagohara et al. (2013). They had suggested that incorporating the iPad and related technological devices into programs for the ASD population might be potentially useful. However, concerns have been raised about the lack of information on generalization of knowledge in some studies and the potential impact of disruptive behaviors on the effectiveness of tablet-based instruction.

Similarly, computer-based interventions also yielded positive results in improving vocabulary in children with ASD. The studies highlighted the importance of factors such as the child’s basic knowledge of computers and the motivation to work with the device. However, the heterogeneity of procedures, samples, and methodologies in studies makes it difficult to draw definitive conclusions about the overall effectiveness of technology-assisted interventions.

When comparing technology-based interventions to nontechnology approaches, the review found that nontechnology interventions, such as instructor-delivered teaching or the use of flashcards, demonstrated more consistent and rapid generalization of vocabulary skills. However, compared to the use of picture books, the use of tablets did not show differences in word learning. Thus, the need for further research is emphasized, particularly in terms of longer-term tablet use and consideration of previous experiences with learning tools, to obtain a more comprehensive understanding of the role of technology in vocabulary development for children with ASD.

In summary, while technology, including tablets and computers, appears to be promising in improving vocabulary skills in some children with ASD, individualized assessments, consideration of previous experiences, and attention to the context of use are crucial. The comparison with non-technology approaches underscores the need for more nuanced investigations to identify the specific conditions under which technology-based interventions can be most beneficial for this population. Future research should provide clearer information on the potential of technology to contribute to the development of vocabulary in children with ASD.

## Data availability statement

The original contributions presented in the study are included in the article/supplementary material, further inquiries can be directed to the corresponding author.

## Author contributions

AU: Conceptualization, Data curation, Formal analysis, Funding acquisition, Investigation, Methodology, Project administration, Resources, Software, Visualization, Writing – original draft, Writing – review & editing. VF-T: Data curation, Investigation, Writing – review & editing. IR-O: Conceptualization, Formal analysis, Funding acquisition, Investigation, Methodology, Project administration, Resources, Supervision, Visualization, Validation, Writing – original draft, Writing – review & editing. DS: Conceptualization, Data curation, Formal analysis, Funding acquisition, Investigation, Methodology, Project administration, Resources, Supervision, Visualization, Writing – original draft, Writing – review & editing.
